# MFL-Based Local Damage Diagnosis and SVM-Based Damage Type Classification for Wire Rope NDE

**DOI:** 10.3390/ma12182894

**Published:** 2019-09-07

**Authors:** Ju-Won Kim, Kassahun Demissie Tola, Dai Quoc Tran, Seunghee Park

**Affiliations:** School of Civil, Architectural Engineering and Landscape Architecture, Sungkyunkwan University, Suwon 16419, Korea (K.D.T.) (D.Q.T.)

**Keywords:** magnetic flux leakage, wire rope non-destructive evaluation, damage type classification, signal-processing, support vector machine

## Abstract

Wire ropes used in various applications such as elevators and cranes to safely carry heavy weights are vulnerable to breakage or cross-sectional loss caused by the external environment. Such damage can pose a serious risk to the safety of the entire structure because damage under tensile force rapidly expands due to concentration of stress. In this study, the magnetic flux leakage (MFL) method was applied to diagnose cuts, corrosion, and compression damage in wire ropes. Magnetic flux signals were measured by scanning damaged wire rope specimens using a multi-channel sensor head and a compact data acquisition system. A series of signal-processing procedures, including the Hilbert transform-based enveloping process, was applied to reduce noise and improve the resolution of signals. The possibility of diagnosing several types of damage was verified using enveloped magnetic flux signals. The characteristics of the MFL signals according to each damage type were then analyzed by comparing the extracted damage indices for each damage type. For automated damage type classification, a support vector machine (SVM)-based classifier was trained using the extracted damage indices. Finally, damage types were automatically classified as cutting and other damages using the trained SVM classifier.

## 1. Introduction

Wire ropes are used in various applications such as elevators and cranes to safely carry heavy weight. However, local damage such as breakage or cross-sectional loss can be caused by the external environment [1].

Damaged parts of the wire rope under tensile force can pose a serious risk to the safety of an entire structure as the damage can rapidly propagate due to concentration of stress. To minimize such risks and accurately diagnose local damage at early stages, a suitable nondestructive evaluation (NDE) technique is required. However, small size damages are not easily detected due to the long length of wire ropes and complex structure of their cross sections. As a result, some wire ropes are used under dangerous conditions [2].

To overcome this situation, a magnetic-sensing-based NDE offers an efficient method of detecting defects in wire ropes because wire rope is made of ferromagnetic materials, which are easily magnetized. In addition, this method is a non-contact NDE, which enables rapid diagnosis. In this method, a wire rope is magnetized using a strong magnetic field, and a magnetic sensor detects variations in the magnetic flux density or leakages of the magnetic field caused by defects in the wire rope [3,4,5].

Among various magnetic NDE options, the magnetic flux leakage (MFL) method is a particularly suitable diagnostic technique for wire ropes due to their continuous ferromagnetic structures. Currently, the MFL technique is widely applied to the diagnosis of various ferromagnetic continuum structures such as pipes, railways, and wire ropes [6,7,8,9]. Most previous studies and techniques using the MFL method have focused on the detection of damages related to cutting, such as cracks and notches [10,11,12,13]. However, in situ wire ropes are vulnerable also to other types of damage, such as cross-sectional loss due to corrosion and shape change due to compression. Although the diagnosis of these types of damage is important, research on the detection of these damage types is lacking. Damage detection and classification of its type are both important for effective management, response, and maintenance.

In this study, the possibility of MFL-based damage detection of various damage types including not only cutting but corrosion and compression were investigated through an experimental study. Furthermore, characteristics of the MFL signals were analyzed to classify damage type.

## 2. Theoretical Background 

### 2.1. Principle of Magnetic Flux Leakage-Based Damage Detection

Wire ropes are generally made using steel materials, which is a ferromagnetic material that can be easily magnetized. When the ferromagnetic wire rope is sufficiently magnetized, the wire rope has the same characteristics as the magnet, and the wire rope and the magnetizing yoke form a closed magnetic path.

When local damage such as cutting or cross-sectional loss of the wire rope occurs, the cross-sectional area through which the magnetic field is passing is reduced at the damaged point. Since the air in the gap of damage cannot have sufficient magnetic field density as the magnet, the magnetic flux in the closed magnetic path leaks at the damaged point to the surrounding space. 

**Figure 1:**
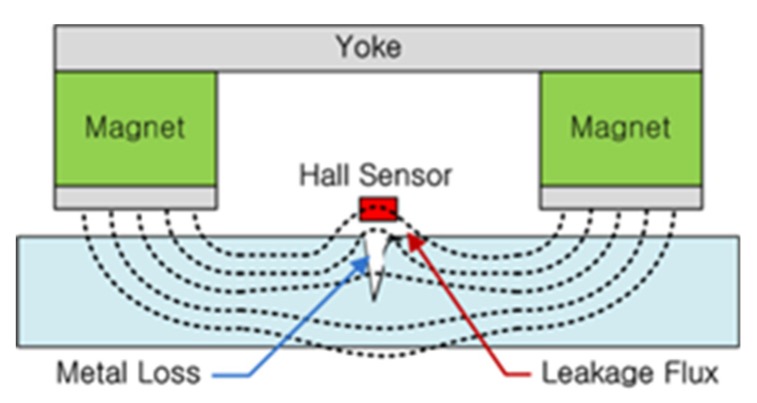
Schematic of the MFL method [14].

To establish magnetic flux in a sample to be diagnosed, a magnetization yoke that can create a strong magnetic field magnetizes the wire rope specimen. If no damage has occurred, the magnetic flux flow in the specimen remains uniform. MFL is generated if local defects are present, as shown in Figure 1 [12,13,14].

**Figure 2:**
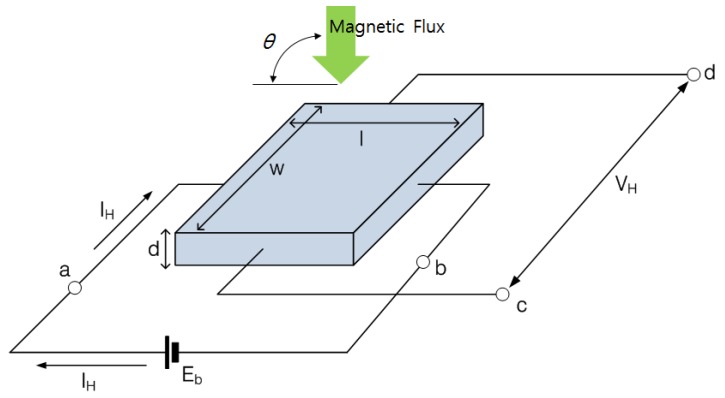
Schematic illustration of the Hall sensor.

An MFL signal can be measured by scanning a specimen which is by measuring the strength of the magnetic field. Magnetic sensors are normally placed between the poles of the magnetization yoke to measure magnetic flux stability. In this study, In-Sb Hall sensor, HW300A model [15], based on the Hall effect was applied to measure the magnetic flux signal. A Hall sensor generates a voltage signal proportional to the magnetic flux [9,12]. Hall sensor is composed of a micrometer order thin film on a semiconductor, and it has electrodes a, b, c, and d, as shown in Figure 2.

When a constant current (*I_H_*) is allowed to flow between the input terminals a–b of the hall sensor and a magnetic field is applied to the Hall sensor, electromotive force is generated between the output terminals c–d by the Lorentz force. Therefore, Hall output voltage (*V_H_*), which is potential difference is generated in proportional to the magnetic flux density *B* [5,9,16]. Since the Hall sensor can only measure the vertical component of the magnetic flux, the Hall output voltage reflects only the vertical component of actual magnetic flux. When the angle between the direction of the actual magnetic flux and the Hall sensor is *θ*, Hall output voltage can be obtained by multiplying the magnetic flux by the sin*θ*. The Hall voltage signal can be measured and filtered and amplified through a data acquisition (DAQ) device. The measured Hall voltage signal indicating the magnetic flux can be used to diagnose the condition of the specimen by analyzing the variations in signal patterns.

### 2.2. Signal Processing and an Enveloping Process Used to Improve the Resolution of MFL Signals

After measuring the magnetic flux signal, de-noising (e.g., low-pass filtering and drift-and-offset correction) was performed to improve the resolution of the signal. First, high-frequency electrical noise was removed by low-pass filtering. The drift and offset were then removed by extracting the low-order mode waveform and subtracting it from the raw magnetic flux signal.

**Figure 3:**
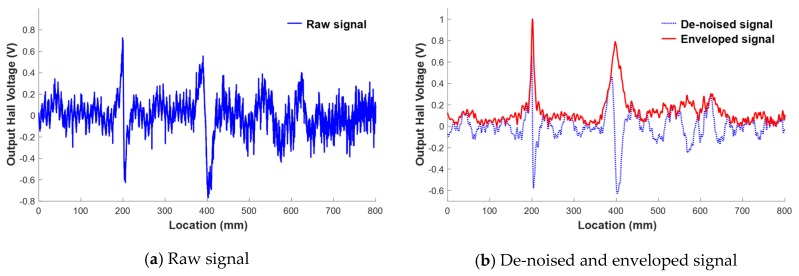
Signal modification observed after the signal processing process. (**a**) Raw signal; (**b**) De-noised and enveloped signal.

After de-noising, a Hilbert transform-based enveloping process was carried out to clarify the flux leakage and improve the sensitivity of damage detection [17]. This enveloping process can reveal important information about the signal by reducing meaningless information such as high-frequency noise. In addition, decision-making in damage detection can benefit from comparing the damage with a threshold value, and various damage index extractions can then be used to quantify the damage level. Signals before and after signal processing are shown in Figure 3.

### 2.3. Visualization Method for Magnetic Flux Leakage Images

Three-dimensional (3D) visualization was applied to create an effective representation of the diagnostic results. Magnetic flux values, measured simultaneously on eight channels, were plotted in a 3D graph. The x-axis is the distance along the wire rope specimen and the y-axis is the measurement channel number that represents the position of the sensor in the circumferential direction. The amplitude of the magnetic flux is color-coded and displayed on the z-axis. 

Magnetic flux signals were visualized in a cylindrical shape, similar to a wire rope. As shown in Equation (1), y- and z-dimensional damage indicators were calculated from the enveloped magnetic flux signals for the visualization process.

*Y_DI_* = (*MF_c_* + r) × cos*θ* *Z_DI_* = (*MF_c_* + r) × sin*θ* (1)

**Figure 4:**
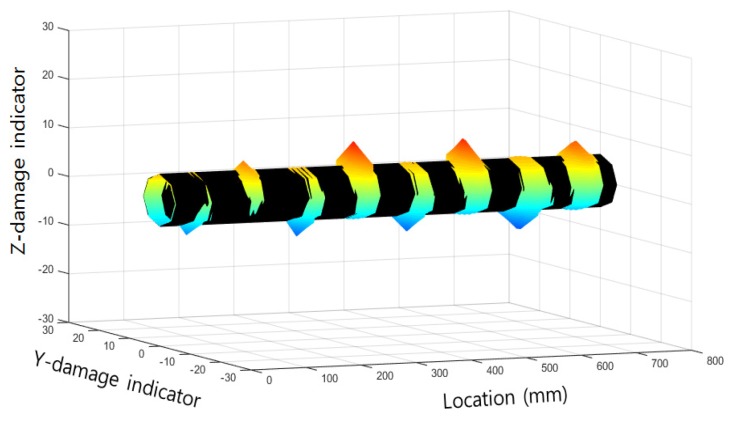
A sample of 3D magnetic flux graph.

*Y_DI_* and *Z_DI_* are the y- and z-dimensional damage indicators, respectively, *MF_c_* is the magnetic flux from the wire rope, *r* is a virtual radius used for wire rope visualization, and *θ* is the circumferential angle of the location of each sensing channel [14]. These calculated indicators were plotted on a 3D graph with a distance indicator. In addition, the 3D magnetic flux graph was wrapped with a cylindrical threshold represented in black. A sample of 3D magnetic flux graph is displayed in Figure 4.

This 3D magnetic flux graph plots the damage location information in the longitudinal and circumferential directions and shows the level of damage from any angle. Damaged areas are highlighted by the threshold covering process. The circumferential direction and damage size can be represented more efficiently by using cross sections of the 3D magnetic flux graph.

### 2.4. Support Vector Machine-Based Automated Damage Type Classification 

In this study, the learning-based support-vector machine (SVM) pattern recognition technique is applied for automatic classification of wire rope damage types. SVM is an automated learning system that classifies the characteristics of training samples by optimal separating hyperplane in high-dimensional feature space [18]. Hyperplane, the decision boundary, is set to have the maximum margin from each group for effective classification, which is called maximal margin SVM.

However, when the hyperplane is linear, it generally has a high misclassification rate when applied to nonlinear samples. Therefore, a nonlinear SVM technique, that determines an optimized nonlinear hyperplane by mapping a two-dimensional feature space into a high-dimensional feature space is required for high accuracy classification.

An optimized nonlinear hyperplane can be obtained by using kernel function that is defined as $K\left(x,x_{i}\right)=(Ф\left(x\right)\cdot Ф\left(x_{i})\right)$ [19]. In this study, polynomial function, one of the representative kernel functions used for nonlinear SVM, is applied to train a SVM classifier.

## 3. Experimental Study 

### 3.1. Experimental Set-Up 

**Figure 5:**
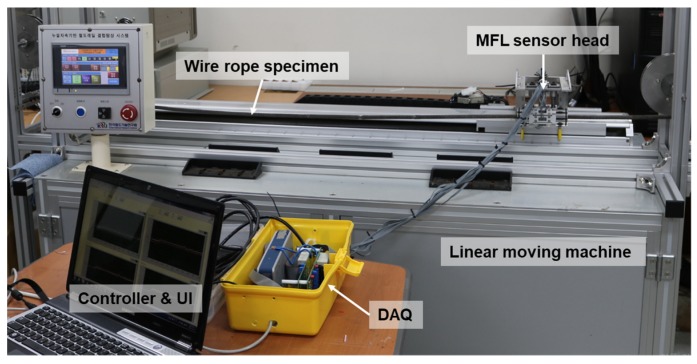
Experimental set-up for wire rope diagnosis.

A series of experimental studies were carried out to examine the diagnostic possibilities for the various types of damage. A linear moving machine capable of moving the sensor head at constant speed was prepared and a damaged wire rope was fixed to its top. To obtain magnetic flux signals, the experimental set-up was composed of a multi-channel MFL sensor head to scan the magnetic flux of each wire rope specimen and a DAQ equipment with a terminal board to operate the Hall sensors, as shown in Figure 5.

**Figure 6:**
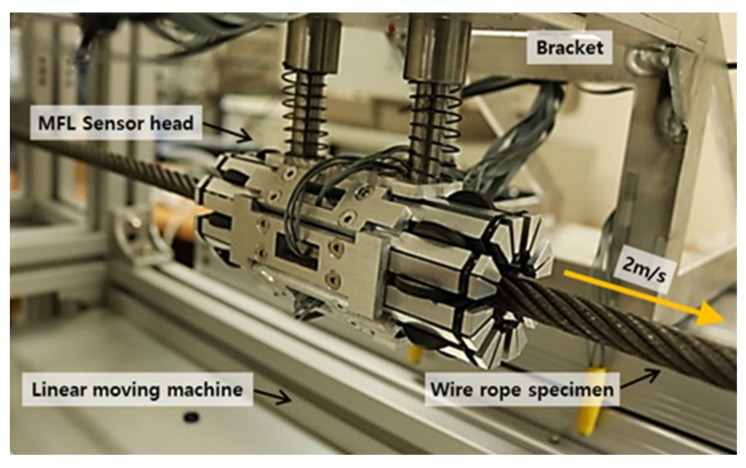
Multi-channel MFL sensor head.

A multi-channel MFL sensor head was fabricated by assembling eight independent sensing modules, each of which was composed of a Hall sensor and a neodymium magnet yoke, that can independently measure magnetic flux signals. These eight modules were circumferentially arranged in a circular configuration to measure eight magnetic flux signals simultaneously from the entire cross section of the wire rope [20,21], as shown in Figure 6.

The eight sensing modules were connected using elastic rubber O-rings to maintain a constant lift-off, which is important for constant sensitivity [22]. This method of joining can also help protect the sensor head by preventing impacts caused by the variable diameters and sudden changes in a specimen by using the flexible expansion and contraction motion of independent sensing modules [21].

In order to obtain magnetic flux signals from various types of damage, three steel wire rope specimens (10 mm in diameter and 800 mm in length) were machined to contain cutting, corrosion, and compression damages.

The linear moving machine was used to scan the fixed wire rope while moving at a constant speed. The MFL sensor head was connected to a bracket of the linear moving machine using a linear bushing and moved along the fixed wire rope specimen at a constant velocity of 2 m/s to measure magnetic flux signals. A set of compact DAQ devices was used to measure the eight magnetic flux signals simultaneously via eight sensing modules as the MFL sensor head moved. The sampling rate was 10 kHz, and signals were measured 40 times along each specimen. Signal processing for noise reduction and the enveloping process based on the Hilbert transform were performed to facilitate effective damage detection.

### 3.2. Cutting Damage Detection Using the MFL Method

**Figure 7:**
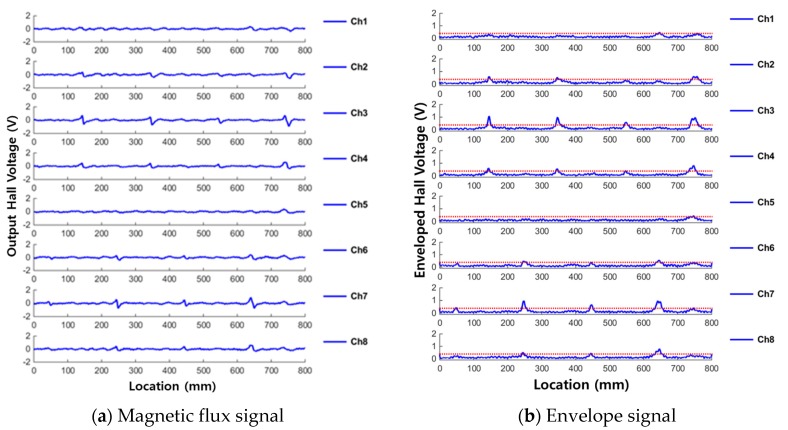
MFL and enveloped signals for detecting cutting damage. (**a**) Magnetic flux signal; (**b**) Envelope signal.

For comparison with the results measured from the corrosion and compression damage shown in Section 3.3 and Section 3.4, the diagnostic results of the cutting damage that was performed by our previous work in reference 20 are summarized and the measured signals are displayed in Figure 7.

In [20], magnetic flux signals, which are measured in the form of output Hall voltage by using DAQ, were measured from several artificial cutting damages formed on wire rope specimens. The measured magnetic flux signal and the enveloped signal after signal processing are displayed in Figure 7.

Figure 7 shows that MFL signals were generated at locations corresponding to the actual damage was location, and enveloped signals exceed the threshold at the damaged points. This shows that detection of cutting damage in a wire rope is possible using the MFL signal.

In addition, the most sensitive sensing channel for damage was the one nearest to the damaged point in the circumferential direction. In addition, lower level of MFL signals were detected in the ambient sensing channels. This shows that the sensitivity for cutting damage detection can be improved when the distance between the damage and the sensing channel is made smaller. Therefore, it is shown that the circumferential direction of damage can be estimated using this NDE method in cutting damage case.

### 3.3. Corrosion Damage Detection 

#### 3.3.1. Test Set-Up and Specimen for Corrosion Damage Detection 

**Figure 8:**
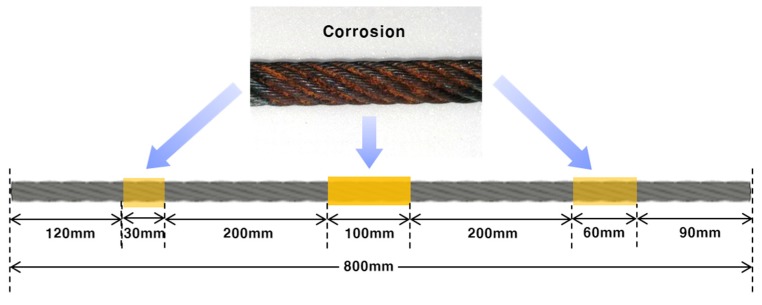
Wire rope specimen with corrosion damage.

For the MFL measurement experiment used for corrosion diagnosis, the same set-up that was used to detect cutting damage was employed. Two identical wire rope specimens with three corroded areas were prepared, as shown in Figure 8.

A 100 mm long segment of corrosion damage was formed at the mid-point (400 mm) of a wire specimen after being wetted by a chemical reagent for 10 days. Additionally, 30 mm and 60 mm long segments of corrosion were formed on either side of the specimen, 250 mm from the center, by being wetted by a chemical reagent for five days, as shown in Figure 8.

In the specimen with corrosion damage, unlike the specimen with cutting damage, it was difficult to specify the sensing channel due to the difficulty of quantitative precision processing. No quantitative index could be used to measure the progress of corrosion, and only the ability to detect damage could be judged. The sampling rate was 10 kHz, the sensor head moved at a speed of 2 m/s, and the signal was measured 40 times.

#### 3.3.2. Test Results for Corrosion Damage Detection 

**Figure 9:**
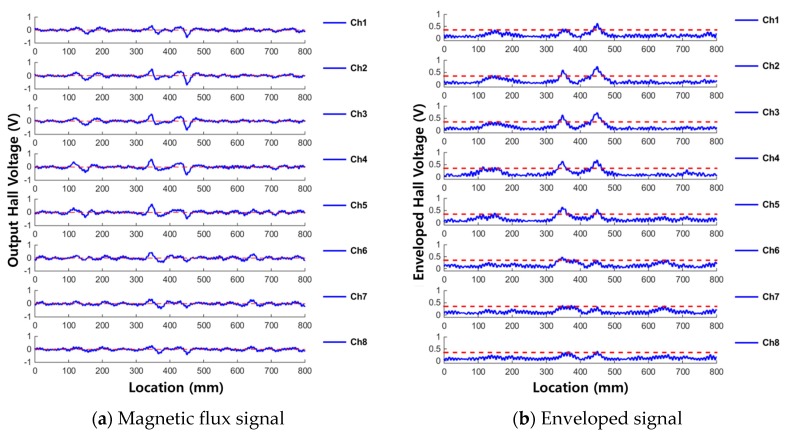
Measured signals of corrosion specimen #1. (**a**) Magnetic flux signal; (**b**) Enveloped signal.

**Figure 10:**
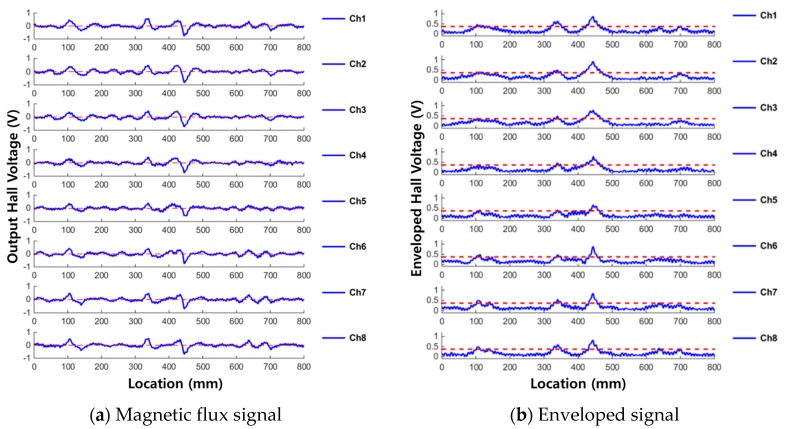
Measured signals of corrosion specimen #2. (**a**) Magnetic flux signal; (**b**) Enveloped signal.

MFL measurement results from the two corroded specimens are shown for each channel in Figure 9 and Figure 10. Figure 9 shows the measured results from corrosion specimen #1 and Figure 10 shows the measured results from corrosion specimen #2. In each figure, the leakage flux signals are shown in Figure 9a and Figure 10a and the enveloped signals are shown in Figure 9b and Figure 10b.

The shapes of the signals are different, but the following common results can be seen in both Figure 9 and Figure 10. First, the 10-day corroded, 100 mm long damaged area (at the 400 mm point) was clearly damaged because the envelope value exceeded the threshold value at the actual damaged point. However, magnetic flux was generated at the beginning and end of the corrosion, unlike the case of cutting damage. MFL signal generally occurs when the cross-sectional area of magnetic flux pass-through changes. Therefore, due to the corrosion damage’s characteristic of the large distance between the start point and the end point of the damage, the magnetic fluxes leaked at both ends are detected separately.

In addition, because the reduction of the cross section was slight, the amplitude of the MFL signals was not large, relatively wide and gentle peaks were generated at the corroded point.

The leaking magnetic flux signal of the first corroded area (at approximately the 140 mm point) exceeded the threshold by slightly different values in some sections and some channels. 

However, for the third corroded area (at approximately the 680 mm point), MFL signal of just over the threshold in magnitude was detected in some channels only in the case of corrosion specimen #2, but none of the channels could detect the damage in the case of corrosion specimen #1; the measured flux was lower in magnitude than the threshold in all channels. 

**Figure 11:**
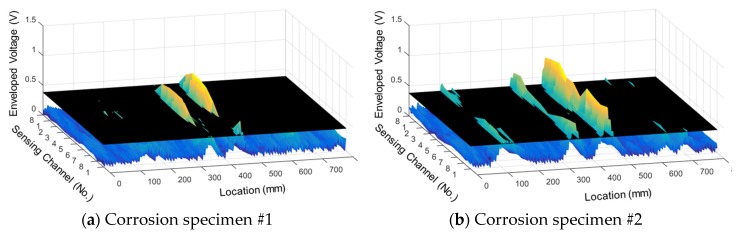
3D visualization of corrosion detection results. (**a**) Corrosion specimen #1; (**b**) Corrosion specimen #2.

In the case of MFL signals detected from corrosion damage, even if the magnitude of the peak was not large, the MFL signals were detected across all channels (as opposed to a specific measurement channel) to show the shape of the corrosion damage. This can be seen in the above results and Figure 11.

As it was confirmed that leaking magnetic flux signals can be measured together in all channels, it appears that corrosion damage can be detected effectively using the sum of the total magnetic flux of all channels. Magnetic flux signals from all each sensing channel were summed to calculate the total flux value. 

**Figure 12:**
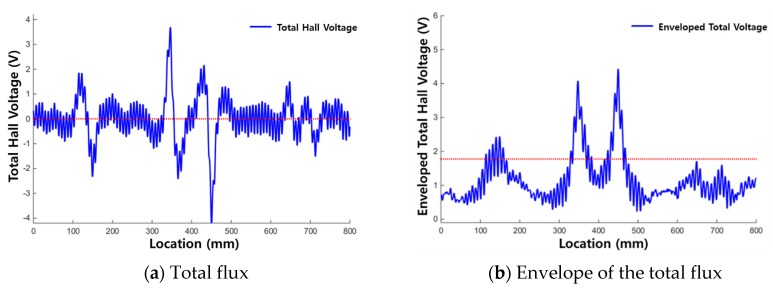
Test results for corrosion specimen #1 using total flux. (**a**) Total flux; (**b**) Envelope of the total flux.

**Figure 13:**
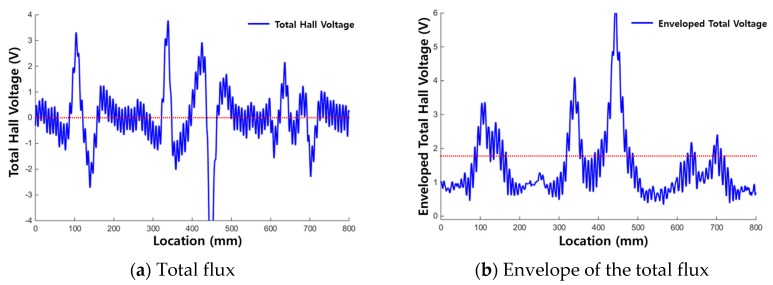
Test results for corrosion specimen #2 using total flux. (**a**) Total flux; (**b**) Envelope of the total flux.

Figure 12a and Figure 13a show the raw signals of the total flux measured from each corrosion specimen, and Figure 12b and Figure 13b show the envelopes of the total flux with the calculated threshold values of the total flux indicated.

When using the total flux signal as shown in Figure 12 and Figure 13, the magnitude and range of the signal exceeding the threshold are larger and more clear than when using the magnetic flux signal of each sensing channel. Therefore, corrosion damage can be detected more clearly by using total flux.

In the results for corrosion specimen #1 (Figure 12), the first and second corroded areas were detected using the total flux method. This is similar to the results when signal of each channel was used discretely. However, the third corroded area was not detected.

In the diagnosis results of corrosion specimen #2 (Figure 13), the third corroded area was detected applying the total flux method. Therefore, determining damage with the total flux signal is useful for the diagnosis of corrosion damage; the magnetic flux signal measured in a single channel may be weak, but MFL signals can be detected uniformly in all channels.

Through the above procedure, it was confirmed that cutting damage and corrosion damage can be diagnosed with the MFL method. An MFL signal was measured at the starting point and end point of a damaged area. 

It is assumed that the surface was oxidized by the progression of corrosion and that the magnetic component of the corroded section changed and divided into separate magnetic sections. Alternatively, an MFL signal may have been caused by a change in the roughness at the surface, where minute damages are emerge.

The MFL signal generated by corrosion had a small peak and a relatively gentle slope. In addition, MFL signals were uniformly measured across all channels, rather than being sensitively measured by a particular channel, confirming that the total flux method is effective for diagnosing corrosion damage. 

### 3.4. Compression Damage Detection 

#### 3.4.1. Test Set-Up and Specimens for Compression Damage Detection

Shape collapse damage occurs when the cross section of a wire rope deforms without breaking. This can be caused by excessive loads when driving the wire rope, mechanical abnormalities, and external impacts. However, a wire rope is usually under a constant load during use and collapse damage may not be visible, making it impossible to visually detect this type of damage during use. In addition, because there is no change in the cross-sectional area of the wire rope, there should be no theoretical change in magnetic flux. It is therefore generally expected that this type of damage cannot be diagnosed using MFL-based NDE equipment and little research has been conducted to confirm the diagnostic possibility of collapse damage.

**Figure 14:**
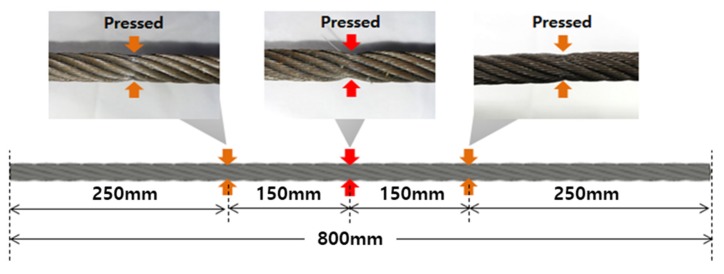
A wire rope with compression damage.

This study examined the possibility of damage diagnosis of compression damage for various types of collapse damages. To prepare a specimen damaged by compression, three kinds of crushing damage were formed on a wire rope (800 mm in length, 10 mm in diameter), as shown in Figure 14.

Compression damage was formed at 250, 400, and 550 mm along the wire rope; these are referred to as compression damages no. 1 to no. 3, from the left to the right, respectively. To form compression damage, the wire rope was crimped in the up and down directions so that deformation would occur only in the shape of the cross section without any loss of the wire. Compression damage no. 1 had a flatness of 82%, with a minimum diameter of 9 mm and a maximum diameter 11 mm. Compression damage no. 2 had a flatness of 67%, with a minimum diameter of 8 mm and a maximum diameter of 12 mm, and compression damage no. 3 had a flatness of 75%, with a minimum diameter of 9 mm and a maximum diameter of 12 mm. The sensor head had moved at a constant speed of 2 m/s and the magnetic flux signal was measured 40 times at a sampling rate of 10 kHz.

#### 3.4.2. Test Results of Compression Damage Detection

**Figure 15:**
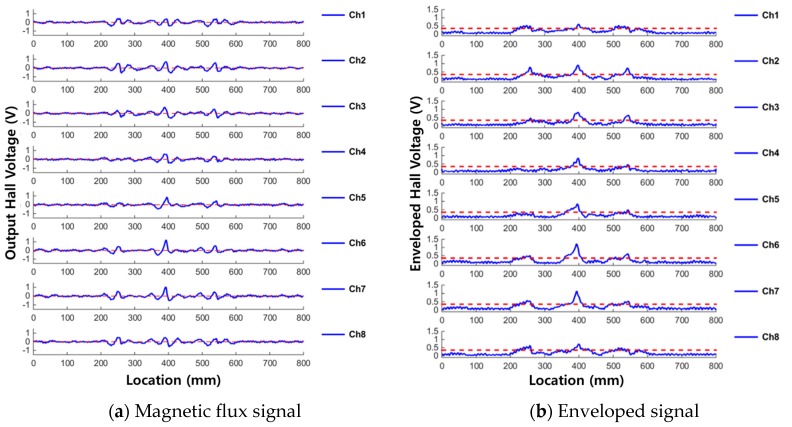
Results of compression damage detection from each sensing channel. (**a**) Magnetic flux signal; (**b**) Enveloped signal.

The test results for compression damage detection are displayed in Figure 15. Figure 15a shows the magnetic flux signals from each sensing channel, and Figure 15b shows the corresponding envelope signals.

**Figure 16:**
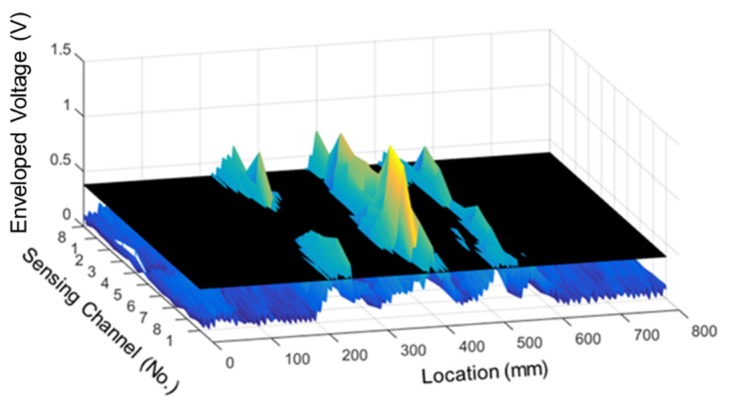
3D visualization of compression damage detection.

MFL signals were observed at 250, 400, and 550 mm along the rope, corresponding to compression damage areas 1, 2, and 3 respectively. Enveloped signals at all three damaged points exceeded the threshold value, and therefore compression damages could be detected. In addition, the largest leakage flux was shown at area 2, which corresponds to the greatest pressed damage. This demonstrates the possibility of quantifying the amount of compression damage. Looking at the shape of the leakage signal in the magnetic flux signal, a W-shaped MFL signal appeared around the center of the pressed damage. This is in contrast to the corrosion damage case, in which the MFL signal appeared at both ends of the damage. In addition, as shown in Figure 16, the distribution of the results for each channel shows that the leakage flux was detected simultaneously in all channels, as was the case with corrosion damage.

**Figure 17:**
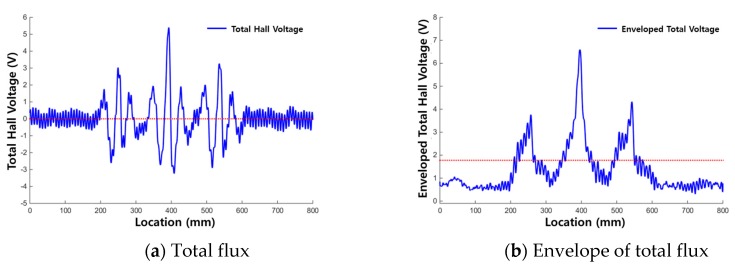
Test results for compression specimen using the total flux. (**a**) Total flux; (**b**) Envelope of the total flux.

The total flux was obtained by the same method as the corrosion damage case, as shown in Figure 17.

Figure 17 shows that MFL signals appeared at all the three compression damage points, and the width of the MFL signal was as wide as 50–100 mm, unlike the case of cutting damage. When compared with the compression damage detection results using the signal of each channel, the ability to detect compression damage was the same for both total flux and the individual channels. However, when analyzing the damage detection for each channel, the MFL signal for compression damage nos. 1 and 3 exceeded the threshold value by slightly different values. Detection results based on the total flux confirmed that the compression damage was clearly detected. Therefore, it can be assumed that the total flux method is necessary to detect compression damage in a sample with more than 80% flatness.

In general, detection of magnetic flux leakage should be difficult in the case of compression damage according to the basic principles of the MFL method. Because, in order for the MFL to occur, the cross-sectional area that the magnetic flux passes through must be changed. However, in the case of compression damage in this study, only the shape of the cross-section changed and there was no change in the cross-sectional area.

However, an MFL signal was detected in the above experiment, likely because magnetic flux leaking from the surface was measured apart from the MFL signal. The magnetization intensity of the system was oversaturated above the knee point (i.e., the ideal saturation). An instantaneous change occurred in the measured value of the magnetic flux signal due to the change in magnetization intensity caused by the change of lift-off. This was due to the momentary upward and downward movement of the sensor module when passing through an area that has experienced crushing damage.

### 3.5. Analysis of Signal Characteristics According to Type of Damage

The possibility of damage detection using the MFL technique was investigated for damage types commonly experienced by wire ropes: cutting, corrosion, and compression damages. According to the experimental results, the MFL method can detect all the three types of damage. However, the MFL signal of each type of damage was slightly different. The characteristics of the MFL signal according to the damage type are summarized below.

**Figure 18:**
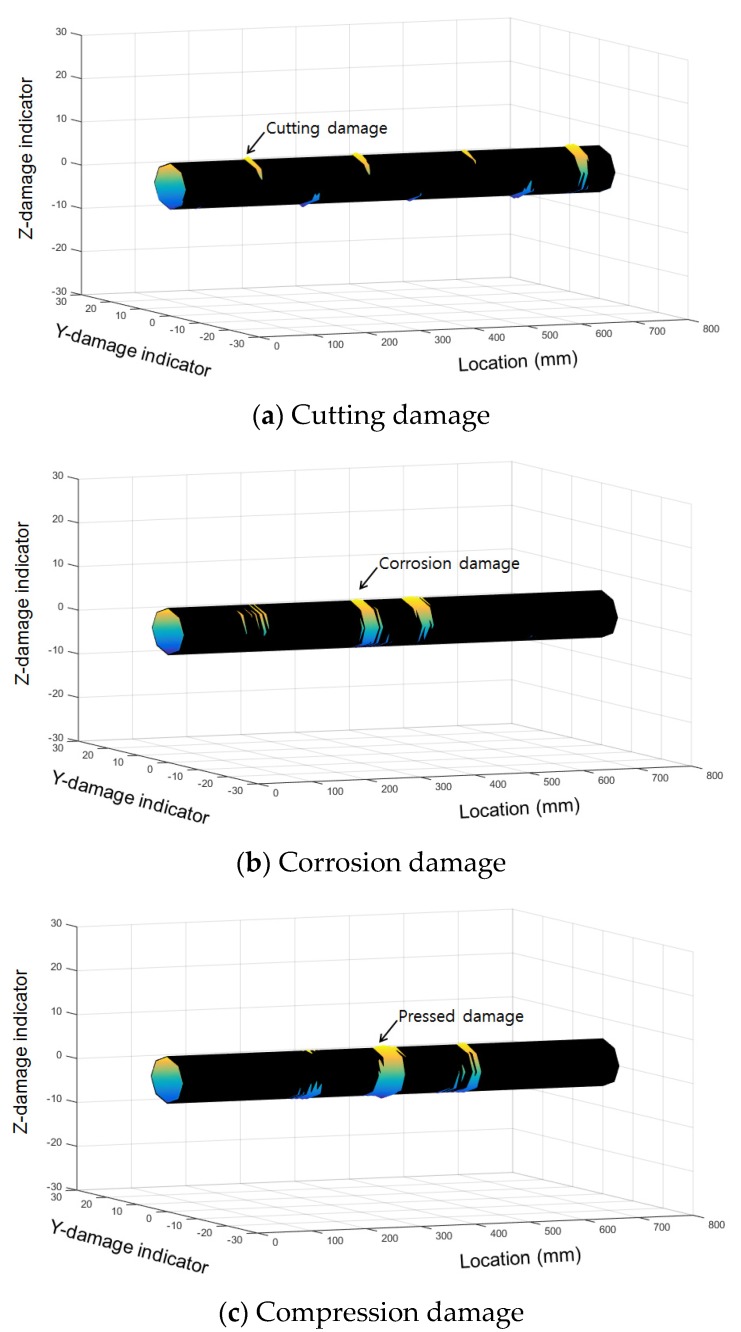
3D visualized images of the test result according to damage type. (**a**) Cutting damage; (**b**) Corrosion damage; (**c**) Compression damage.

**Figure 19:**
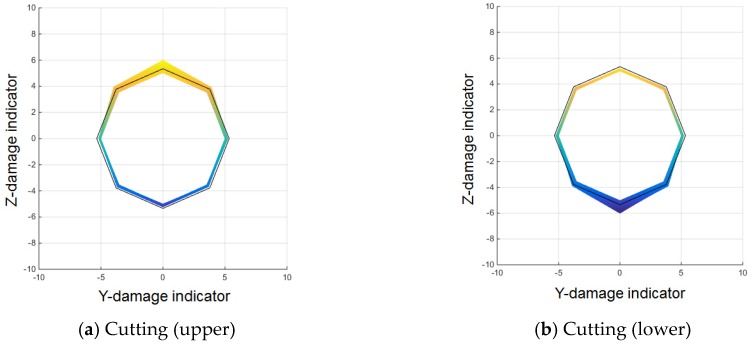

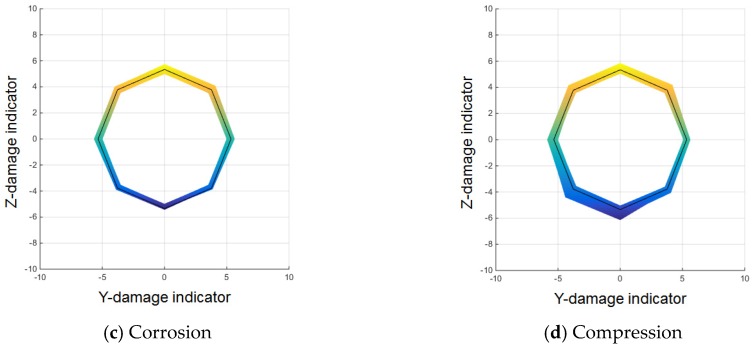
Cross-sectional magnetic flux distribution at the damaged point according to damage type. (**a**) Cutting (upper); (**b**) Cutting (lower); (**c**) Corrosion; (**d**) Compression.

For the purpose mentioned, the measured MFL signals from corrosion, compression, and cutting (with a cut depth of 0.5 mm) are compared. First, as shown in Figure 18 and Figure 19, signals from the cutting damage case and the other damage cases differ in the sensing channel where damage is detected.

For cutting damage, as shown in Figure 18a and Figure 19a,b, the MFL signal was measured sensitively only in a specific sensing channel in the direction in which the damage was located. Even if a large sized of damage occurred, higher peaks were observed only in the channels close to the damaged area; peaks in channels that were farther away remained relatively low.

This response could change if the cutting damage occurred in the entire circumferential direction, but the probability of such damage in real wire ropes is low, and this study did not consider that type of multiple and complex damage.

For corrosion and compression damage, similar, relatively low leakage fluxes were detected in all sensing channels for both types of damage. Figure 18b and Figure 19c show the results of the corrosion damage diagnosis, and Figure 18c and Figure 19d show the results of the compression damage diagnosis.

**Figure 20:**
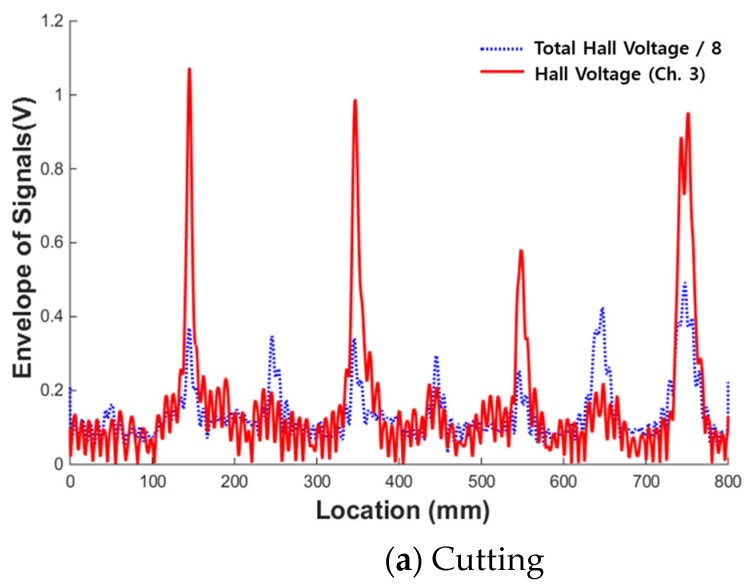

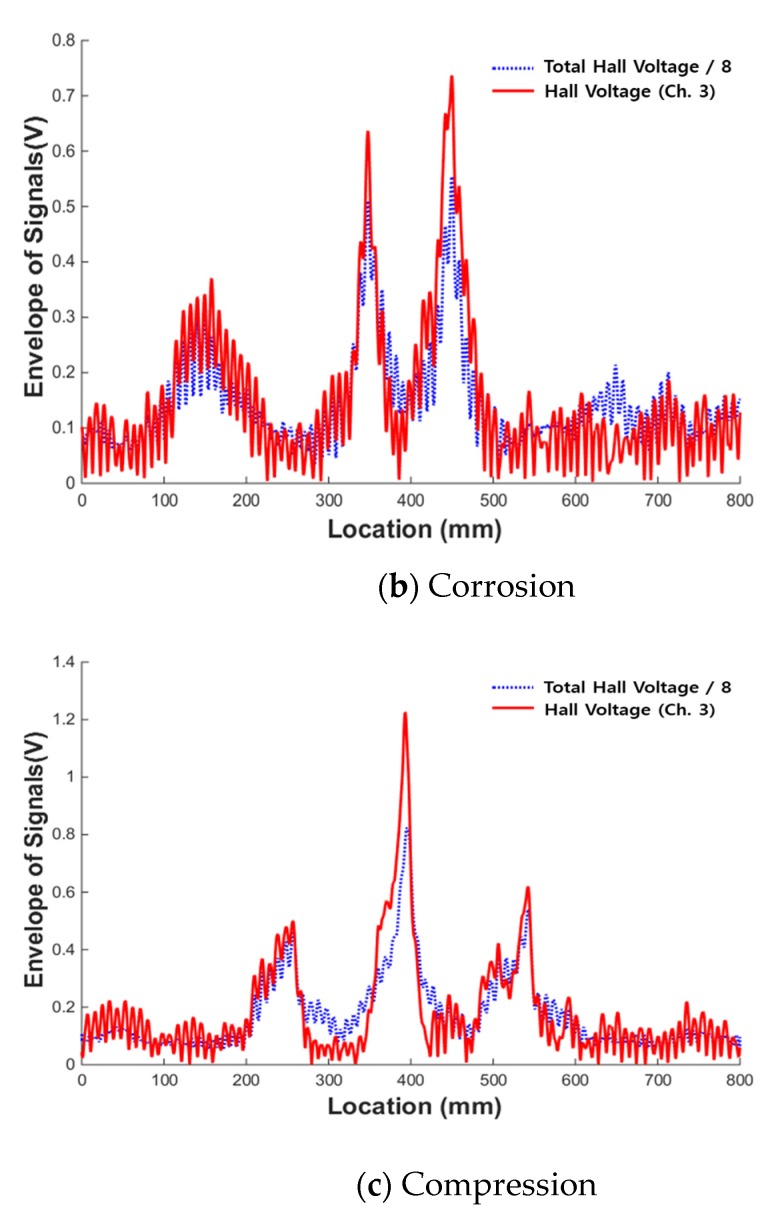
Comparison of the magnetic flux of a single channel and the total flux. (**a**) Cutting; (**b**) Corrosion; (**c**) Compression.

To utilize these characteristics, the ratio between the MFL magnitude of a single channel where the maximum peak appeared and the total MFL magnitude of all the channels was applied as an index. The average magnetic flux signal was obtained by dividing the total flux by the number of sensing channels in the sensor head. To distinguish differences by type of damage, the magnetic flux signal where the maximum peak appeared and the average magnetic flux signal were compared, as shown in Figure 20.

First, a comparison of the maximum magnetic flux signal detected by sensing channel #3 and the average magnetic flux signal in the case of the cutting damage shown in Figure 20a indicates that the magnitude of the peak of sensing channel #3 was at least 2–3 times larger than the peak of the average magnetic flux signal. If the value of the corresponding channel is deducted when calculating the average magnetic flux value, the magnification can be further increased.

Figure 20b illustrates the case of corrosion damage. The ratio of the maximum peak to the average peak is 1.5 or less. The same is true for the compressive damage case, as shown in Figure 20c. The value of the magnetic flux of a single channel divided by the total flux can therefore be used as an index to differentiate between cutting and the other damage types.

To quantify this characteristic, two kinds of indices were extracted. The peak of the single channel was divided by the sum of the flux values of the remaining seven channels to calculate the peak/total-7 ratio (PT7).

Moreover, when the size of the damage was large, MFL signals could be detected even in the multiple sensing channels near the damage due to the blooming phenomenon. To maximize the ratio of the total flux to a single peak, the total flux 5 through the sum of the magnetic flux from five channels excluding three channels near the maximum peak detected was calculated. The maximum peak was then divided by the total flux 5 value to calculate the peak/total-5 ratio (PT5).

**Figure 21:**
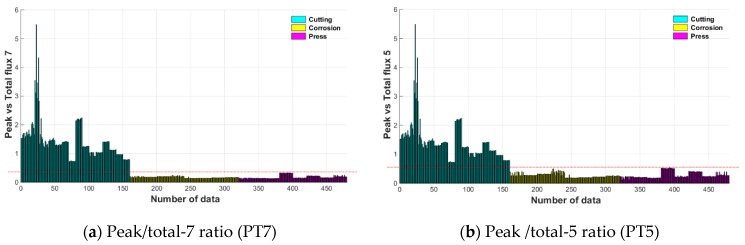
Peak/total ratio indices according to damage type. (**a**) Peak/total-7 ratio (PT7); (**b**) Peak /total-5 ratio (PT5).

Figure 21 shows PT7 and PT5 values extracted from 160 peaks for each damage type. The PT7 and PT5 values are high only in the case of cutting damage, and values for corrosion and compression damages are low. The value of the magnetic flux of a single channel divided by the total flux can be used as an index to differentiate between cutting and the other damage types. These indices can be used as a parameter in the damage classifier based on the pattern recognition technique.

**Figure 22:**
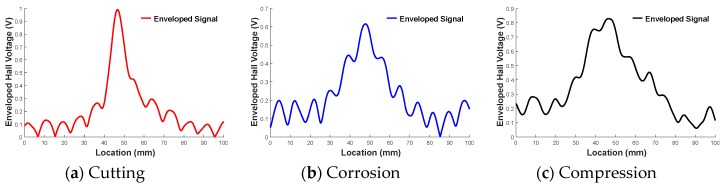
MFL peak shape according to damage type. (**a**) Cutting; (**b**) Corrosion; (**c**) Compression.

To analyze the characteristics of individual MFL peaks, examples of single MFL peak from each type of damage are displayed in Figure 22.

By comparing the peaks of the cutting, corrosion, and compression damages, it becomes clear that the peak from cutting damage was narrower and larger in magnitude than the peaks associated with other damage types. This becomes even more apparent as the depth of the cutting damage gets increased. Corrosion and crushing produced shorter and wider peaks.

**Figure 23:**
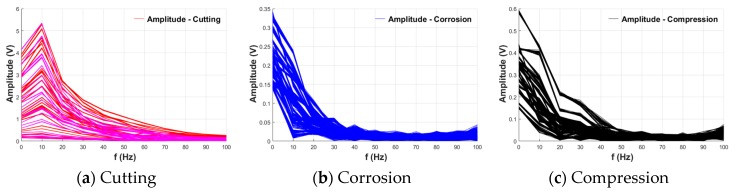
Power spectra of peaks according to damage type. (**a**) Cutting; (**b**) Corrosion; (**c**) Compression.

Due to the above characteristics, the frequency characteristics of the MFL peak were also affected according to the damage type. To analyze this, the power spectrum of the peak of each damage type was obtained through fast Fourier transform (FFT). These results are plotted repeatedly in Figure 23 [23,24].

As shown in Figure 23, the frequencies were not divided in detail because they were converted using a short range (1000 points) compared with the sampling rate (10 kHz). However, it was confirmed that cutting and other damage types have different power spectra. For the other damage types, maximum amplitudes were common in the frequency range converging to 0 Hz. On the other hand, for cutting damage, the maximum amplitude did not converge to 0 Hz, and appeared near 10 Hz in most cases. A maximum amplitude at a relatively high frequency in the frequency domain means that there can be a large number of peaks in the same sampling point, indicating that the slope of the peak is relatively steep. These results indicate that the frequency characteristics of the MFL peak can be used as an index to identify the type of damage.

**Figure 24:**
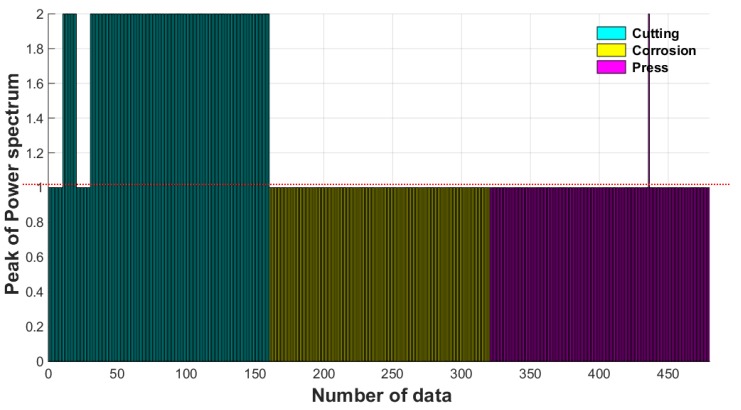
Maximum peak of the power spectrum.

The position of the maximum peak is indexed to levels and shown in Figure 24. The convergence to zero in Figure 23 is indexed to level 1 and the case of 10 Hz to level 2.

As shown in Figure 24, most cases of cutting damage have a value of 2, and most others have a value of 1, which can be used as a damage index for classification. However, a value that can be misinterpreted as the other type of damage is found in the extraction value of some rounds, it shows that the level is 1 in a part where the peak in magnitude is very small. Therefore, an independent index alone cannot completely classify damages, and it is necessary to compensate by combining it with other indices.

By comparing the characteristics of the MFL signals for each type of damage, several features were found to distinguish cutting from the other types of damage. The usable features were the number of simultaneous detection channels at the time of damage detection and the ratio of the height and width of the peak. These were quantified by determining the magnetic flux of a single channel divided by the total flux and the frequency range of the maximum amplitudes in the power spectra, respectively. By using these indices, it is expected that the damage type can be separated into two types: cutting and other types of damage. Based on the relevance of the accident occurrence, the scope of this study was limited to classifying the damage type into cutting and others (including corrosion and compression).

### 3.6. Support Vector Machine-Based Damage Type Classification

To train a classification to automatically classify the damage type as cutting or others, a support vector machine (SVM)-based pattern recognition technique was applied. When the magnetic flux signal measured through the sensor head exceeded the set threshold value, the part was regarded as damage. A search for the channel on which the largest MFL signal is measured was then made. Damage indices were extracted based on the magnetic flux signal of the channel.

**Figure 25:**
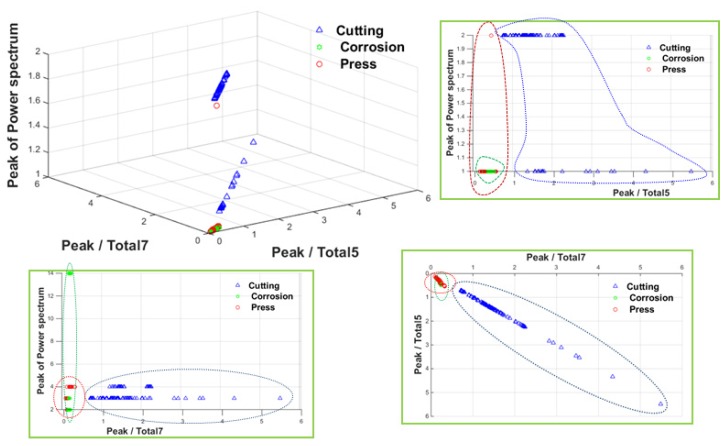
Three-dimensional learning data distribution for classification of damage type.

Using the three kinds of extracted damage indices as parameters, the SVM-based pattern recognition algorithm classified the type of damage. In this study, PT7, PT5, and peak of power spectrum were used as parameters to train the SVM. Three kinds of indices for training data set extracted from 160 peaks for each type of damage are shown as a 3D graph in Figure 25.

**Figure 26:**
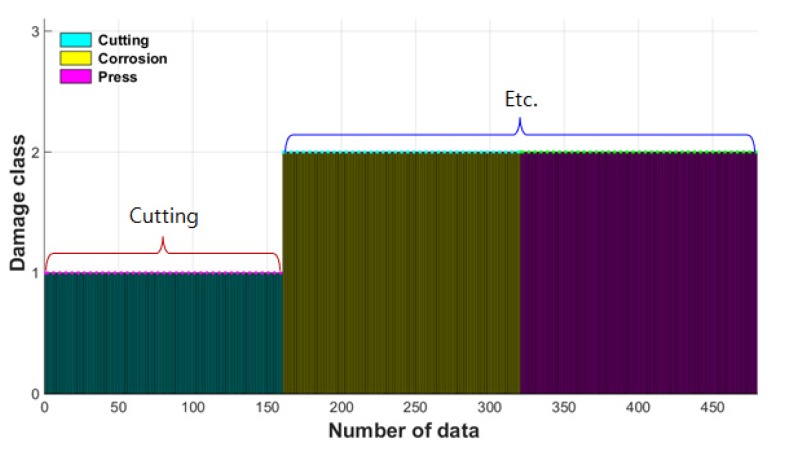
Damage type classification result using SVM classifier.

The SVM classifier was trained based on the above set of damage indices, and the trained SVM classifier was used for the automatic damage classification for wire rope NDE. To verify the performance of the trained SVM classifier, a new data set collected in the same measurement conditions and times was classified through the trained SVM classifier. The label for cutting damage was set to 1, the label for other damages was set to 2, and the classification results are shown in Figure 26.

A total of 480 newly measured MFL peak signals from damage point were classified using the trained SVM classifier, which classified the damage types of 160 as cutting damage and 320 as other damages with 100% accuracy. We therefore concluded that the damage type of wire rope can be effectively estimated by classifying the damage type automatically using the trained classifier based on SVM pattern recognition.

## 4. Conclusions

In this study, three types of damage commonly experienced by wire ropes (cutting, corrosion, and compression) were diagnosed using the MFL technique. This was done through a series of experiments, and the characteristics of the MFL signals in accord with to each damage type were analyzed. The possibility of objective damage diagnosis using threshold values was confirmed, and various characteristics of the MFL signal corresponding to damaged areas were analyzed.

When detecting cutting damage, MFL signals caused by cutting damage were detected clearly only in sensing channels close to the damaged points. Peripheral sensing channels also detected damage when the size of the damage expanded. On the other hand, in the damage case of corrosion and compression, low-level amplitude MFL was detected evenly in all the sensing channels. It is therefore advantageous to detect corrosion and compression damage using total flux, which is the sum of flux leakages of all channels.

Using these characteristics of MFL signals, a damage index was proposed to classify damage type; this index is defined as the ratio between the peaks of a single sensing channel and total flux.

In addition, cutting and other damage types also differed by the slope of the peak in the MFL signal; the position of the peak of the power spectrum in the frequency domain can be extracted through FFT analysis to quantify a peak’s slope.

The support vector machine method using the three indices extracted was applied to automatically classify the damage type. When classifying the newly measured MFL signals using the trained classifier, the classifier estimated the type of damage as cutting and other damage types with 100% accuracy.

Overall, these results demonstrated that the proposed MFL- and SVM-based damage detection and classification method is able to diagnose damages and to estimate the damage type in the wire ropes used in lifting structures.

However, further studies are needed on more various types of damages for the in situ application of the proposed method. In addition, a pattern recognition algorithm that can quantify the damage size will need to be studied for an automated damage analysis.

It is expected that the MFL-based automated NDE technique can be utilized as an advanced inspection tool for a reliable remote wire rope monitoring system through the convergence with various smart technologies.
